# Degradation of D-2-hydroxyglutarate in the presence of isocitrate dehydrogenase mutations

**DOI:** 10.1038/s41598-019-43891-3

**Published:** 2019-05-15

**Authors:** Raffaela S. Berger, Lisa Ellmann, Joerg Reinders, Marina Kreutz, Thomas Stempfl, Peter J. Oefner, Katja Dettmer

**Affiliations:** 1 Institute of Functional Genomics, University of Regensburg, Am BioPark 9, 93053 Regensburg, Germany; 2 Present Address: Leibniz Research Centre for Working Environment and Human Factors, Ardeystr. 67, 44139 Dortmund, Germany; 3 Department of Hematology and Medical Oncology, University of Regensburg, Franz-Josef-Strauss-Allee 11, 93052 Regensburg, Germany; 4 Center of Excellence for Fluorescent Bioanalytics, Am BioPark 9, 93053 Regensburg, Germany

**Keywords:** Metabolomics, Cancer metabolism, Enzyme mechanisms

## Abstract

D-2-Hydroxyglutarate (D-2-HG) is regarded as an oncometabolite. It is found at elevated levels in certain malignancies such as acute myeloid leukaemia and glioma. It is produced by a mutated isocitrate dehydrogenase IDH1/2, a low-affinity/high-capacity enzyme. Its degradation, in contrast, is catalysed by the high-affinity/low-capacity enzyme D-2-hydroxyglutarate dehydrogenase (D2HDH). So far, it has not been proven experimentally that the accumulation of D-2-HG in *IDH* mutant cells is the result of its insufficient degradation by D2HDH. Therefore, we developed an LC-MS/MS-based enzyme activity assay that measures the temporal drop in substrate and compared this to the expression of D2HDH protein as measured by Western blot. Our data clearly indicate, that the maximum D-2-HG degradation rate by D2HDH is reached *in vivo*, as v_max_ is low in comparison to production of D-2-HG by mutant IDH1/2. The latter seems to be limited only by substrate availability. Further, incubation of IDH wild type cells for up to 48 hours with 5 mM D-2-HG did not result in a significant increase in either D2HDH protein abundance or enzyme activity.

## Introduction

Among the oncogenic mutations reported to alter cancer cell metabolism, mutations in isocitrate dehydrogenase (*IDH*), which normally catalyses the oxidative decarboxylation of isocitrate to α-ketoglutarate (αKG), have attracted particular attention^1,2^. Missense mutations in both cytosolic *IDH1* and mitochondrial *IDH2* are identified frequently in grade II and III astrocytomas and oligodendrogliomas, secondary glioblastomas, and acute myeloid leukaemia (AML)^3,4^. The mutations result in a neo-enzymatic activity, *i.e*. the production of D-2-hydroxyglutarate (D-2-HG), but not L-2-HG^5,6^, from αKG, accompanied by an accumulation of the product^5^. Increased D-2-HG levels, which can be as high as 35 mM in glioma tissue^5^, are associated with promotion of oncogenesis and inhibition of differentiation. Due to its structural similarity to αKG, D-2-HG has been shown to exert inhibitory effects on α-ketoglutarate-dependent enzymes^7–9^, which depicts one possible way of mediating metabolic transformation. Additionally, Sulkowski *et al*. (2017) reported, that elevated concentrations of 2-HG cause a homologous recombination (HR) defect^10^.

D-2-HG is also produced physiologically in low amounts as a by-product of hydroxyl acid-oxoacid-transhydrogenase (HOT)-enzyme activity^11^. There is no specific physiological role of D-2-HG known, but it is sufficiently oxidized back to αKG by a specific dehydrogenase located in the mitochondrion. There exists one enzyme for the degradation of each enantiomer, D-/L-2-hydroxyglutarate dehydrogenase (D-/L-2HDH)^12,13^. Deficiencies of D2HDH or L2HDH due to germline or post-zygotic mutations result in 2-HG aciduria (2-HGA type I), which is associated with encephalopathy^14–16^. Reduced expression of L2HDH has been also observed in renal cancer^17^, but it causes only an about 10-fold increase in tumour levels of L-2-HG. In contrast, mutations in *IDH1/2* increase D-2-HG levels by two to three orders of magnitude. Therefore, it is hypothesized that D-2-HG production by mutated IDH1/2 exceeds the degradation capacity of functional D2HDH^18,19^. This situation is similar to D-2-HGA type II, where a germline mutation in *IDH2* is present. In these patients, similar to cancer, *IDH2* mutation leads to the production and accumulation of D-2-HG in the presence of functional D2HDH, which results in an even higher urinary excretion of D-2-HG compared to patients with D-2-HGA type I^20^. This also suggests that D-2-HG production exceeds its degradation and excretion.

We have implemented an HPLC-MS/MS-based assay to measure D-2-HG production and degradation in cells to investigate, for the first time, D-2-HG degradation under elevated D-2-HG levels as a result of mutations in *IDH1/2*. We compare the D-2-HG degradation capacities of several cell lines under various conditions. For cells producing D-2-HG, we hypothesized that the degradation rate of D-2-HG is increased to meet the elevated D-2-HG levels. Such enzyme regulation can be achieved at several levels and, therefore, we measured in addition to the enzyme activity of D2HDH also its abundance by Western blot. The results contribute to a better understanding of the mechanism of D-2-HG accumulation in tumours with *IDH1/2* mutations.

## Methods

### Cell culture and lysis

The human breast cancer and fibrosarcoma cell lines MCF7 (ATCC HTB-22, ATCC, Manassas, Virginia) and HT1080 (ATCC CCL-121) were cultured in DMEM (PAN, Aidenbach, Germany) supplemented with 10% fetal calf serum (Biochrom AG, Berlin, Germany), 1% penicillin-streptomycin (PAA Laboratories Inc., Pasching, Austria), and 2 mM L-glutamine (PAA). The human acute lymphoblastic leukemia cell line CCRF-CEM-C7H2 (kindly provided by R. Kofler, Innsbruck, Austria) and various clones of the human colon cancer cell line HCT116 (HD 104-013, HD 104-019, HD 104-020, Horizon Discovery, Waterbeach, UK) were cultured in RPMI1640 (PAN) supplemented with 10% fetal calf serum (Biochrom AG) and 2 mM L-glutamine (PAN). Adherent cells were sub-cultured by trypsinization followed by centrifugation for pelleting, while suspension cells were collected simply by centrifugation (5 min, 200 × *g*).

### D-2-HG dehydrogenase assay

Cell pellets were washed twice with phosphate buffered saline (PBS; PAN) and resuspended in assay buffer (20 mM HEPES, 25 mM KCl, 0.05 mM ZnCl_2_, protease inhibitor cocktail (cOmplete EDTA-free, Roche, Basel, Switzerland). Sonication (3 × 10 sec at 90%, Bandelin Sonopuls, HD2070, Bandelin electronic GmbH&Co. KG, Berlin, Germany) was performed on ice. Centrifugation at 10,000 × *g* at 4 °C for 5 min removed cellular debris. The cell lysate supernatant was used for the dehydrogenase assay and kept on ice for a maximum of 6 h until the assay was started by the addition of PMS (Sigma Aldrich, Taufkirchen, Germany) and D-2-HG (Sigma Aldrich). PMS was added after an aliquot of the cell lysate had been taken for protein quantification. For assaying dehydrogenase activity, the cell lysate supernatant (200–400 µL, larger volumes for more time points) was incubated with increasing concentrations of D-2-HG at 37 °C and gentle shaking (400 rpm). Aliquots of 20 µL were taken over a time period of up to 5 h, stable isotope-labelled standard (2,3,3-d_3_-2-HG, C/D/N Isotopes Inc., Pointe-Claire, Canada; 100 μM in water) was added, before samples were immediately quenched in 100 µL of cold methanol to stop the reaction. After extraction, samples were analysed by LC-MS/MS (see below). For calculation of rate and K_m_ values, only data points within a linear range of degradation were taken into account. Replicates (n = 3) of cells from two different passages were lysed and used for assay experiments.

### D-2-HG formation assay

D-2-HG formation was measured using a protocol adapted from Pusch *et al*.^21^. Briefly, cells were prepared as described for the D2HDH assay and lysed by sonication (3 × 10 sec at 90%, Bandelin Sonopuls, HD2070) in assay buffer (50 mM Tris-HCl, pH 7.4, 10 mM NaCl, 2 mM MgCl_2_, protease inhibitor cocktail (Roche)). The assay was started by the addition of different concentrations (100 µM, 1 mM, 5 mM, 10 mM) of αKG and 10 mM NADPH. Collection and extraction of aliquots were performed as described for the D2HDH assay.

### Sample preparation for 2-HG quantification

For determination of intracellular 2-HG levels, adherent cells were washed twice with PBS before 10 µL of stable isotope-labelled internal standard solution (2,3,3-d_3_-2-HG, C/D/N Isotopes Inc., Pointe-Claire, Canada; 100 μM in water) were added to cells, which were then scraped in 600 µL of 80% cold methanol. Suspension cells were also washed twice with PBS followed by addition of internal standard as before and precipitation in 600 µL of 80% cold methanol. For complete extraction, the sample was centrifuged at 95,600 × *g*, (4 °C, 5 min) and the supernatant was collected. The pellet was washed twice with 100 µL of 80% methanol and supernatants were combined in a glass vial. Samples were dried using an infrared vortex vacuum evaporator (CombiDancer, Hettich AG, Baech, Switzerland) and reconstituted in 50 µL of deionised water. The same sample preparation steps were applied to assay aliquots. Metabolite concentrations were normalized to protein content.

### 2-HG quantification by HPLC-MS/MS

HPLC-MS/MS analysis was performed (as described recently)^22^ using an Agilent (Boeblingen, Germany) 1200 Series HPLC system coupled to an API 4000 QTRAP (AB SCIEX, Darmstadt, Germany) mass spectrometer operating in negative ionization mode. A Discovery HS F5-3 HPLC column (15 cm × 2.1 mm, 3 µm; Supelco, Bellefonte, PA, USA) equipped with a Security Guard column (C18, Phenomenex, Aschaffenburg, Germany) was used. Gradient elution was performed as described in Supplementary Table S5 with phase A consisting of 0.1% formic acid (FA) in water (v/v) and 100% acetonitrile (ACN) as mobile phase B. The column was kept at 30 °C, and an injection volume of 5 μL was used. Turbo ion spray was operated employing the following parameters: gas 1 and 2: 50 psi and curtain gas: 10 psi. The ion spray voltage was set to −4500 V, the declustering potential to −40.0 V, the entrance potential to −10.0 V, the collision exit potential to −5 V, and the collision energy to −24 V. Detection was performed in multiple reaction monitoring (MRM) mode using the following ion transitions: m/z 147.07 (M - H)^−^ to m/z 84.80 for 2-HG and m/z 150.07 to m/z 87.80 for the deuterated internal standard. Quantification was achieved using a calibration curve constructed from the area ratio of 2-HG to the stable isotope-labelled standard.

### Enantioselective 2-HG analysis

For enantioselective analysis of 2-HG, samples were derivatized according to Gibson *et al*.^23^. See also supplement.

### Data analysis

The HPLC-MS/MS software used was Analyst 1.6.2 (AB Sciex). Further data analysis was performed using MS Excel and R (version 3.0.0). For determination of K_m_ Origin Pro (v8.0724, OriginLab Corporation, Northampton, MA, USA) was used.

### Protein quantification

Protein content of cellular lysates used in the dehydrogenase assay and for Western blotting was determined by means of the FluoroProfile® Protein Quantification Kit (Sigma Aldrich). Protein pellets obtained after methanol precipitation were resuspended in 20 mM of sodium dihydrogen phosphate buffer with 1.2% SDS. The samples were diluted when necessary in water.

### Western blot

Cells were lysed in 20 mM sodium dihydrogen phosphate buffer with 1.2% SDS and adjusted to equal protein amounts. Western blots were performed according to standard protocol. Briefly, we used BoltTM Bis-Tris Plus gels (4–12%, Invitrogen/ThermoFisher Scientific, Waltham, MA, USA) for SDS-PAGE. Gels were blotted onto a 0.45 µM PVDF membrane (Immobilon-P, Merck Millipore, Burlington, MA, USA). Membranes were blocked in Roti-Block (Carl Roth, Karlsruhe, Germany) prior to incubation with first antibody (anti-hD2HGDH, Catalogue No. 13895-1-AP, Proteintech Group Inc., Rosemont, IL, USA; 1:500, diluted in Roti-Block) over night. As secondary antibody, HRP-linked IgG (Amersham NA9340V/GE Healthcare, Little Chalfont, UK) was used, diluted in TBS-T (10 mM Tris-HCl, 150 mM NaCl, 0.05% Tween 20, pH 7.4) 1:4500. SuperSignal West Dura (ThermoFisher Scientific) was added 5 min before imaging, followed by image capture using the BioRad VersaDoc imaging system (4000 MP, BioRad Laboratories, Hercules, CA, USA). Coomassie staining was performed for loading control. Relative quantification was performed with ImageLab (version 5.1, BioRad).

### qPCR

Total RNA was isolated from cells using the RNeasy Mini Kit (Qiagen, Hilden, Germany). The Bioanalyzer (Agilent) was used for quality control before cDNA was prepared from 1 µg RNA using SuperScript™ II Reverse Transcriptase (Life Technologies, Darmstadt, Germany). Relative D2HGDH messenger RNA (mRNA) levels were determined by quantitative polymerase chain reaction (qPCR) using a gene-specific TaqMan assay (Hs_00292260) on an ABI Prism 7000 (Applied Biosystems/ThermoFisher Scientific, Waltham, MA, USA). Results were analysed with the Sequence Detector Software (Applied Biosystems) using the delta-delta-CT method.

## Results

### HPLC-MS/MS analysis of 2-HG

**Figure 1:**
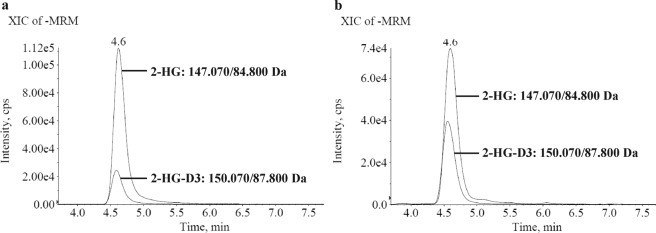
Representative chromatograms of 2-HG and the internal standard 2-HG-d_3_. (**a**) Standard sample (conc[iS] = 10 µM, conc[2-HG] = 14.1 µM), and (**b**) biological sample (cell culture supernatant of HCT116 IDH2R172K, conc[iS] = 20 µM, conc[2-HG] = 14.1 µM). iS = internal standard.

The D2HDH activity assay is based on the HPLC-MS/MS analysis of 2-HG on an achiral column; hence, 2-HG enantiomers are not separated. A representative chromatogram of a standard sample and an extract of a supernatant from HCT116 IDH2-R172K cells is shown is Fig. 1. 2-HG and its deuterated isotopologue elute as symmetrical peaks. For absolute quantification, calibration curves were generated. Figures of merit established with standard solutions in water are presented in Supplementary Tables S1 and S2. The linear range was determined according to the FDA Guide for Bioanalytical Method Validation^24^. Herein, the lower (LLOQ) and the upper limit of quantification (ULOQ) are defined as the lowest, respectively, highest point of the calibration curve with an accuracy between 80% and 120%. With an injection volume of 5 µL, the LOD of 0.05 µM corresponds to 2.5 fmol. Accuracy and precision were calculated from three spike levels (close to LLOQ, medium concentration and close to ULOQ) applied to blank medium and were within the acceptable range of 100% ± 15% (±20% at the LLOQ and ULOQ). Recovery was calculated from media with and without 10% FCS as a protein source and ranged from 101.8% to 125.5%.

### Enzymatic activity assay for D-2-HG degradation

Assays published to date measure D-2-HG degradation either photometrically via the reduction of DCIP (dichlorophenol indophenol) or via the production of labelled L-glutamate from labelled D-2-HG^12,25^. The latter assay, however, may be biased by a lower rate of the second step, namely the conversion of αKG to L-glutamate. For our assay, enantiomerical pure D-2-HG was added to cell homogenates and the decline in 2-HG concentration was analysed quantitatively by HPLC-MS/MS. Enantioselective test measurements of cell extract and assay aliquots showed, that only the D-form of 2-HG was present in the samples and, therefore, we measured indeed only the degradation of D-2-HG (Supplementary Figs S1 and S2).

**Figure 2:**
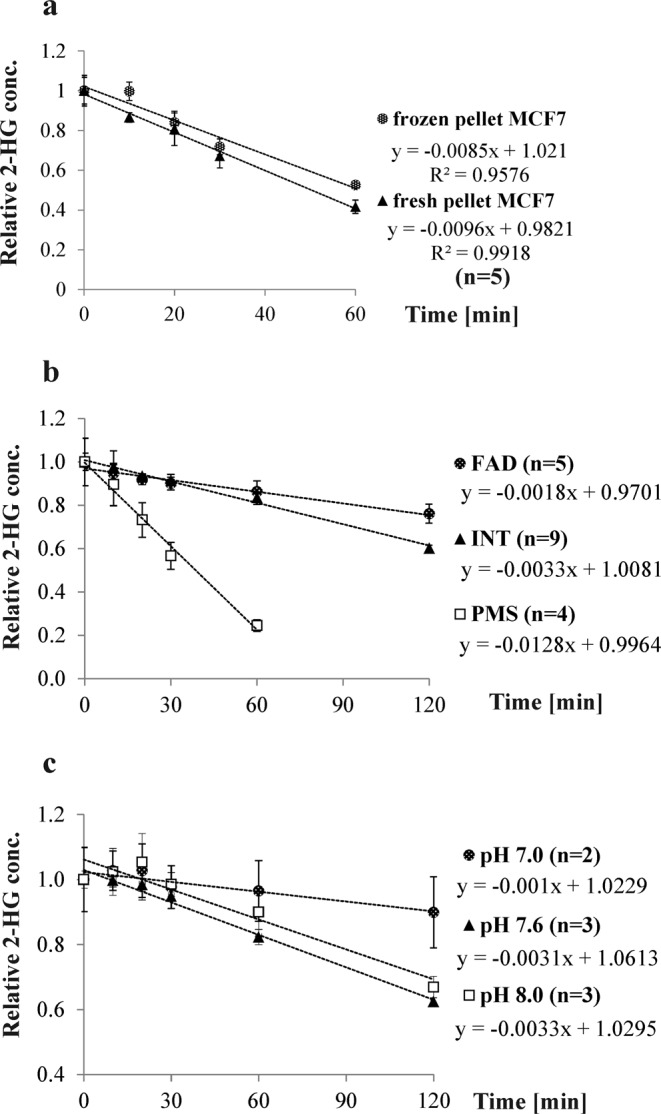
The D2HDH assay was optimized by testing different assay conditions using cell homogenates from MCF7 cells. In all plots, 2-HG concentration normalized to protein content per aliquot was set 100% at t = 0 min. (**a**) Frozen cell pellets yield lower enzymatic rates than freshly prepared cell pellets (n = 5 for both). (**b**) Impact of different redox equivalents: PMS shows a significantly higher degradation rate than FAD and INT (ANOVA: p = 2.04 × 10^−15^). (**c**) pH 7.0, pH 7.6, and pH 8.0 were compared to establish optimal pH conditions for the D2HDH activity assay. D-2-HG degradation rate was highest at pH 7.6 (ANOVA: p = 3.01 × 10^−4^), reflecting mitochondrial pH.

HEPES buffer (20 mM) was used for cell lysis by sonication as described^25^. Cells used for measuring D2HDH activity have to be used directly without freezing to avoid loss of enzyme activity (see Fig. 2a). Since D2HDH oxidizes D-2-HG to αKG, an oxidizing agent must be added to the reaction mix. Different agents have been used as redox equivalents in published assays such as the endogenously utilized flavin adenine dinucleotide (FAD)^12^, iodonitrotetrazolium (INT)^26^, and phenazine methosulfate (PMS)^25^. Therefore, FAD, INT, and PMS were tested and compared to each other using substrate concentrations that yielded a slope of linear degradation. At a substrate concentration of 20 µM D-2-HG, FAD gave the lowest, and PMS the highest D2HDH enzyme activity (see Fig. 2b, ANOVA: p = 2.04 × 10^−15^, TukeyHSD PMS vs INT: p = 3.80 × 10^−14^, PMS vs FAD: p = 3.60 × 10^−14^, FAD vs INT: p = 1.70 × 10^−4^). Therefore, PMS was used as redox equivalent. Furthermore, the impact of pH on D-2-HG degradation was tested by performing the assay at three different pH-values: 7.0, 7.6, and 8.0. Again, by comparing the slopes, D-2-HG degradation was found to be highest at pH 7.6 (see Fig. 2c; ANOVA: p = 3.01 × 10^−4^; TukeyHSD pH 7.6 vs pH 8: p = 9.18 × 10^−3^, pH 7.0 vs pH 7.6: p = 1.74 × 10^−4^, pH 7.0 vs pH 8.0: p = 1.66 × 10^−3^). This corresponds to the pH milieu found in the mitochondrion^27^.

### D2HDH activity in different cell lines

**Figure 3:**
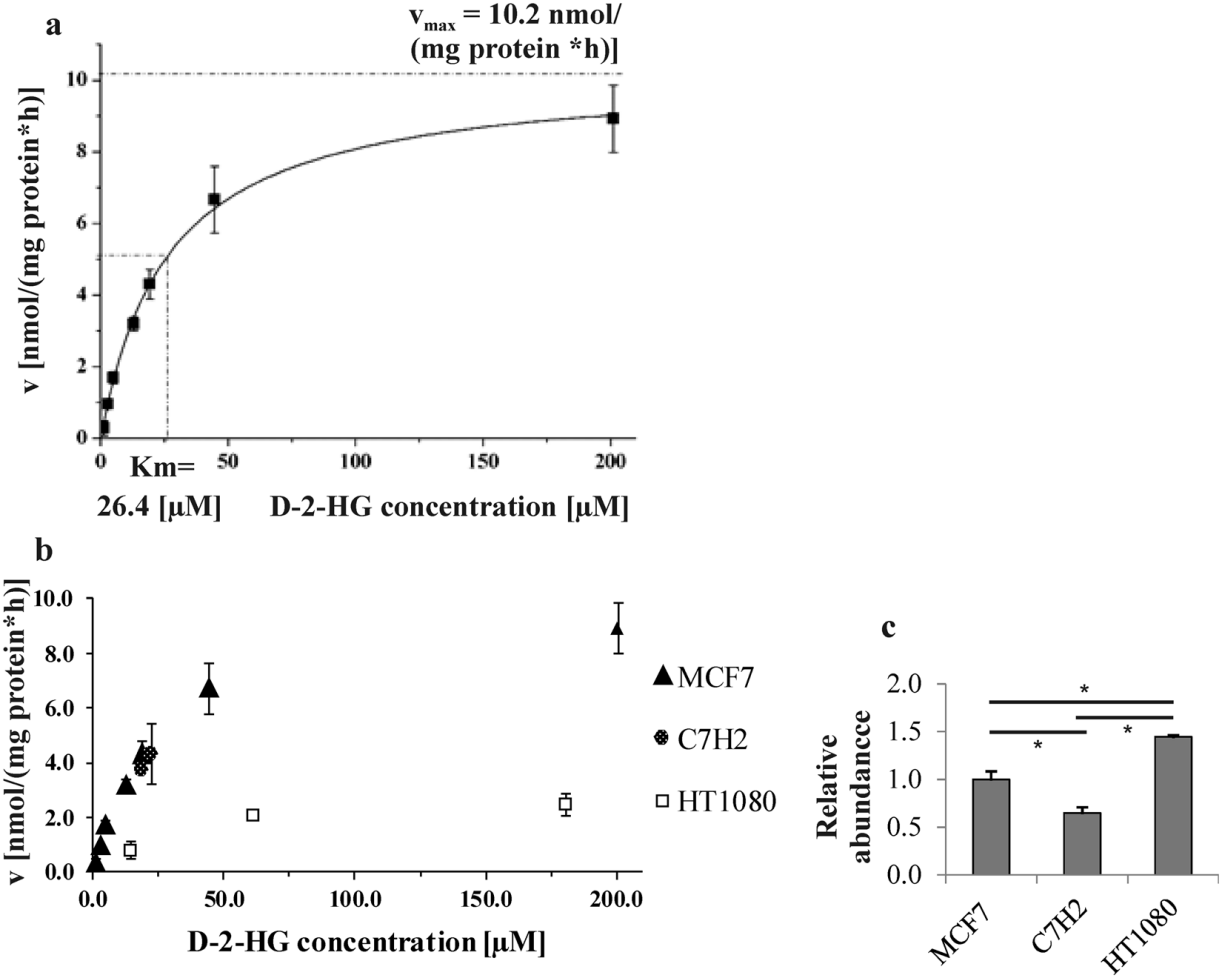
Enzyme activity and protein abundance of D2HDH was tested in three different cell lines. (**a**) Using MCF7 cells, a K_m_ of 26.4 µM (standard error 1.65; n = 3) was determined for D2HDH. (**b**) Comparison of three different cell lines shows D2HDH activity to be lower in HT1080 than MCF7 and C7H2 cells. (**c**) Relative protein abundance for D2HDH (normalized to MCF7) is different for the cell lines tested but does not reflect D2HDH enzyme activity. (n = 3, ANOVA p: 0.0007, TukeyHSD: HT1080 vs. C7H2 p = 8.20 × 10^−6^; MCF7 vs. C7H2 p = 9.23 × 10^−4^, MCF7 vs. HT1080 p = 2.51 × 10^−4^). For Western blot data see also Supplementary Fig. S4.

Using the final assay protocol, the K_m_ value for D-2-HG in MCF7 cells, a human breast cancer cell line, was determined by applying different D-2-HG concentrations to aliquots of the same cellular lysate. By means of this approach the K_m_ of D2HDH in MCF7 cells was approximated to be 26.4 µM (standard error 1.65, R^2^_adj_ = 0.99718; n = 3 per data point, two independent experiments; see Fig. 3a) using the hill function (n = 1 for a single substrate model) in OriginPro 8. Applying the assay to the acute lymphoblastic leukaemia T-cell line C7H2 and the fibrosarcoma cell line HT1080, respectively, showed C7H2 cells to have a D2HDH activity similar to that of MCF7 cells, while HT1080 cells showed a lower activity with a v_max_ of about 3 nmol/(mg total protein × h) (see Fig. 3b). This is especially intriguing, as HT1080 cells carry an *IDH1-R132C* mutation and, therefore, produce high endogenous intracellular levels of D-2-HG that are more than >500-fold higher than those of MCF7 or C7H2 cells (see Supplementary Table S3). To exclude the perturbation of D-2-HG degradation measurements by D-2-HG production within the lysate, stable-isotope labelled αKG was added instead of D-2-HG. Formation of the corresponding labelled D-2-HG by mutated *IDH* was not observed (see Supplementary Fig. S3).

It should be noted that enzymatic rates are normalized to total protein amount in the cell lysate. However, in addition to enzyme kinetics, regulation of enzyme activity may occur by changes in D2HDH protein expression. Furthermore, posttranslational enzyme modifications resulting in altered enzyme characteristics may contribute to differences in degradation rates. Therefore, we analysed D2HDH protein expression by Western blot. The observed differences in maximal degradation capacity were not reflected in D2HDH protein abundance in HT1080 cells (see Fig. 3c). Contrary to the lower degradation capacity, D2HDH protein abundance in HT1080 cells was about 1.45-fold higher compared to that of MCF7 cells (see Supplementary Table S4). Generally, D2HDH protein abundance did not vary much across the cell lines tested, though expression differences were significant for some cell lines (see Supplementary Table S4).

**Figure 4:**
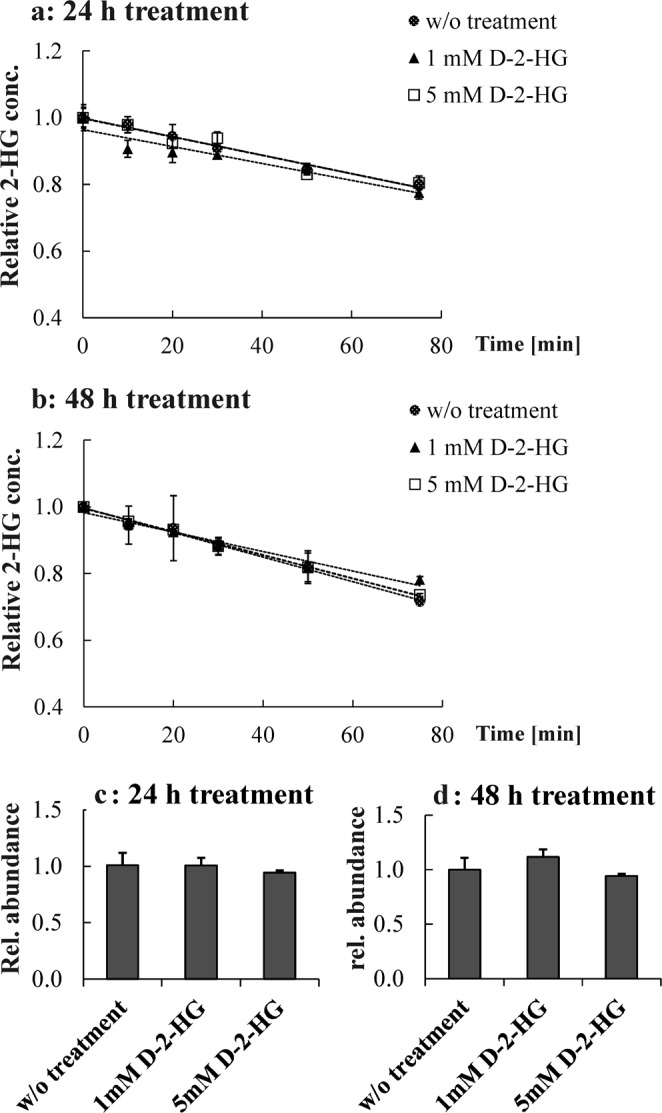
Enzyme activity of D2HDH in MCF7 does not change as a function of D-2-HG concentration and duration of treatment: (**a**) 24 h (ANOVA p = 0.7789) or (**b**) 48 h (ANOVA p = 0.0694) (n = 2 for each time point). D2HDH protein abundance in MCF7 after 24 h **(c**) and 48 h (**d**) of D-2-HG treatment, (n = 3) from Western blot. No significant difference in protein abundance was observed, ANOVA: p = 0.50478 (**c**) and p = 0.06892 (**d**), respectively.

As stated earlier, we expected HT1080 cells to have increased D2HDH activity as a consequence of *IDH1* mutation and high endogenous D-2-HG levels. Apparently, D2HDH expressed in HT1080 cells does not fulfil the expectation of a positive feedback to increase D-2-HG degradation. To the best of our knowledge, these cells do not express mutated D2HDH. Protein abundance was slightly elevated, while isoelectric focusing yielded inconclusive results. Still, we were asking whether D2HDH is regulated in response to D-2-HG levels. Therefore, MCF7 cells characterized by endogenously low D-2-HG levels (0.042 nmol/mg protein ± 18.3%) were treated with 1 mM and 5 mM D-2-HG for 24 h and 48 h. The treatment increased neither enzyme activity nor D2HDH abundance (see Fig. 4). In addition, the impact of 1 mM D-2-HG treatment on MCF7 and C7H2 cells was checked at the *D2HGDH* mRNA-level but was not found to be upregulated (see Supplementary Fig. S5).

**Figure 5:**
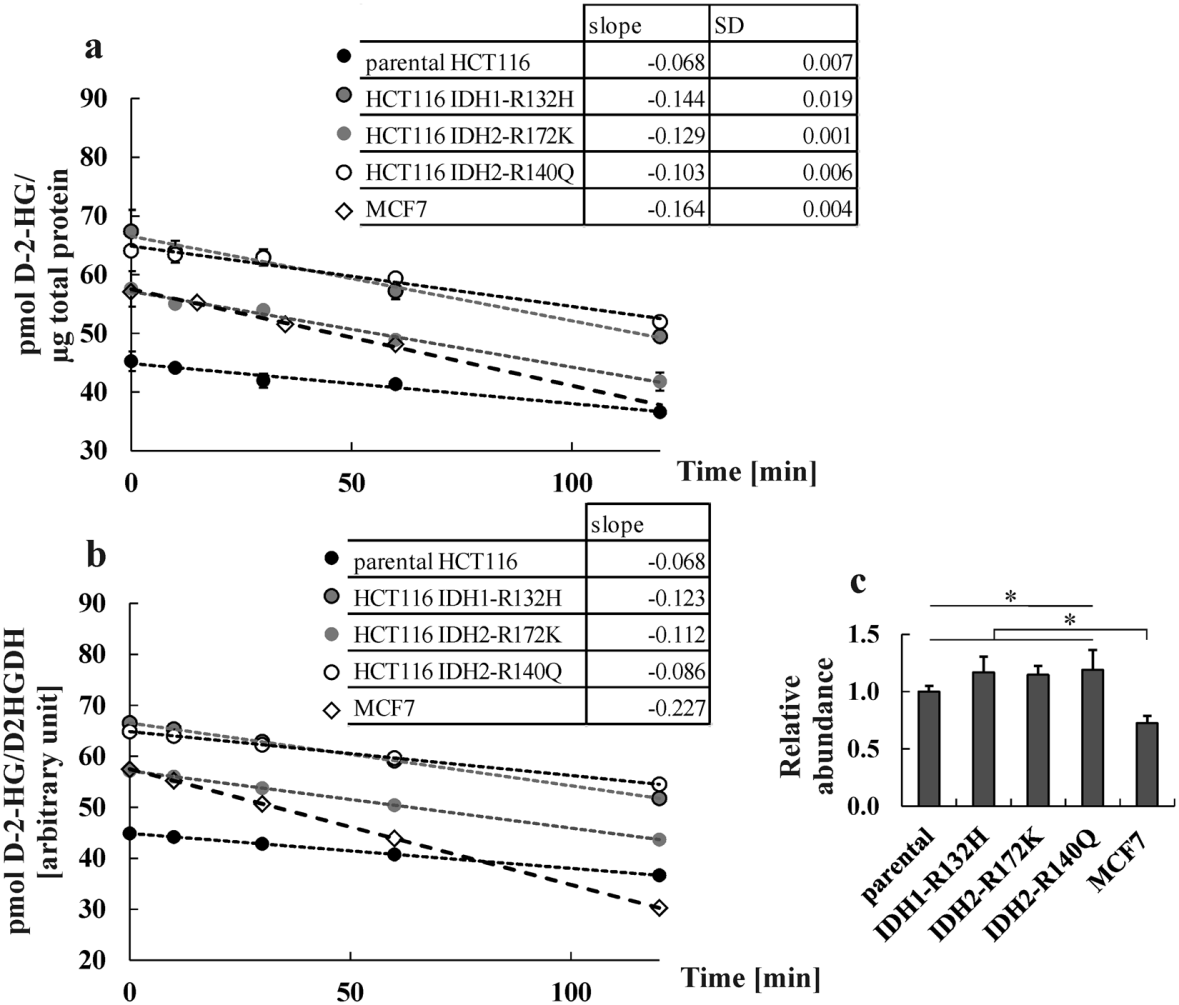
Comparing degradation rates for D-2-HG. (**a**) Degradation rates of D-2-HG by D2HDH in MCF7 and different HCT116 cells are shown at v_max_ of MCF7 (n = 2–3). (**b**) Degradation rates were recalculated correcting for differences in D2HDH protein abundance. (**c**) D2HDH abundance by Western blot in cells of the HCT116 panel and in MCF7 cells were normalized to expression in parental HCT116. Expression differences are significant between parental HCT116 and IDH2-R140Q (TukeyHSD p = 0.0324) and for MCF7 against all HCT116 panel cell lines (n = 3–7, see Supplementary Table S4).

Measuring intracellular 2-HG level in MCF7 cells after treatment showed that uptake of D-2-HG resulted in 2-HG levels that were about two orders of magnitude lower compared to the concentration of 85 nmol 2-HG/mg protein in HT1080 cells. Consequently, these experiments were repeated with the HCT116 cell panel containing a parental cell line and three cell lines with different *IDH* mutations *(IDH1-R132H*/+, *IDH2-R172K*/+, *and IDH2-R140Q*/+). The latter cells carrying *IDH1/2*-mutations produce D-2-HG endogenously, which was confirmed by quantifying intracellular (as well as extracellular) concentrations (see Supplementary Table S3). The parental HCT116 line showed baseline levels that were comparable to those of MCF7 cells. Cells with a mutation had clearly elevated levels. This was particularly true for cells carrying *IDH1-R132H* and *IDH2-R172K* mutations: their 2-HG levels were comparable to those of HT1080 cells. The 2-HG levels of the *IDH2-R140Q* clone was higher than that of parental HCT116 but lower than that of the *IDH2-R172K* clone, which is in agreement to published data^28^. Protein expression, on the other hand, was higher in the parental and *IDH* mutant HCT116 clones relative to the MCF7 cell line (see Fig. 5 and Supplementary Table S4). There is a tendency of higher D2HDH expression in the IDH mutant HCT116 clones compared to the parental clone, but the difference is only significant for IDH2-R140Q (ANOVA p = 0.0275, TukeyHSD par vs R140Q: p = 0.0324, see Fig. 5). Differences in D-2-HG degradation were difficult to detect; most likely, differences in activity were smaller than the assay variance (see Supplementary Fig. S6). When comparing degradation at a concentration reflecting v_max_ of MCF7 of around 50 pmol 2-HG/µg protein, it can be seen that slopes of degradation of HCT116 cells differ (Fig. 5a). These differences could be due to the observed differences in D2HDH protein levels (Fig. 5c and Supplementary Table S4). We recalculated the slopes using the fold changes in D2HDH protein abundance to correct for differential expression (Fig. 5b). Using these data, the degradation rate for MCF7 cells was clearly higher at the chosen concentration, while degradation rates among the HCT116 clones differed only slightly.

### Comparing production of D-2-HG by mutIDH versus its degradation by D2HDH

**Figure 6:**
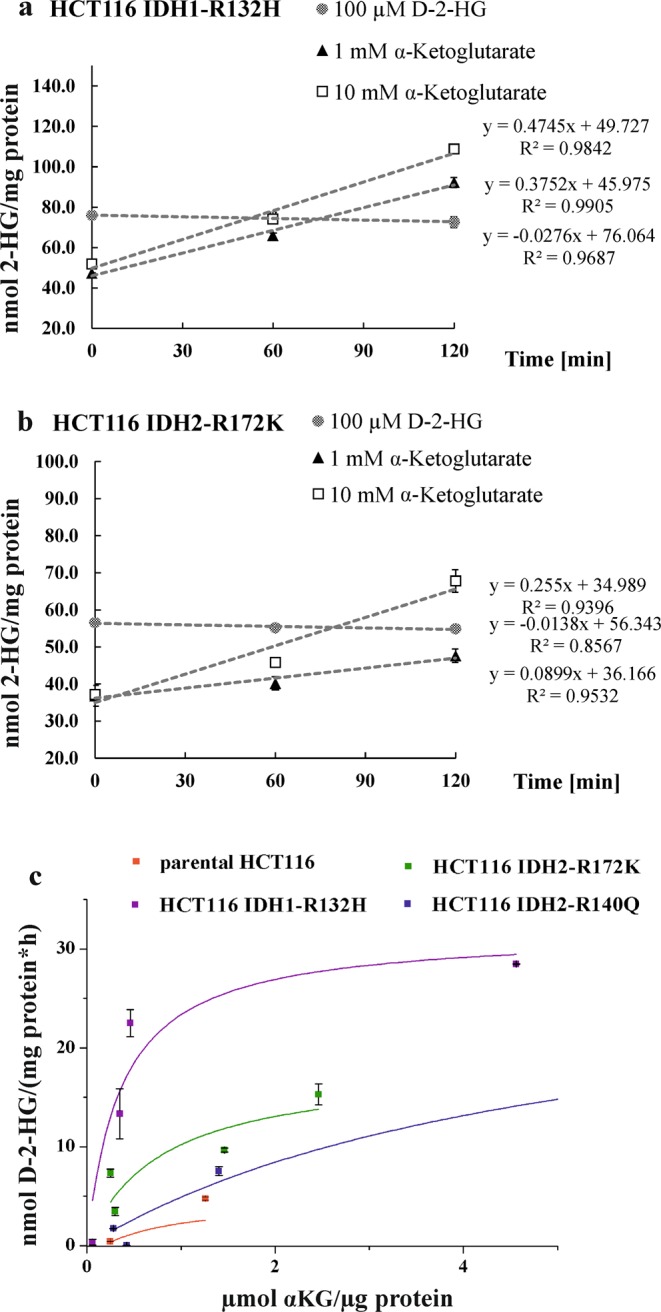
Comparison of D-2-HG formation by mutated IDH to D-2-HG degradation by D2HDH in cell-based enzyme assays in (**a**) HCT116 IDH1-R132H cells and (**b**) HCT116 IDH2-R172K cells (n = 2): Degradation capacity is found low and is not further increased at higher substrate concentrations. In this assay degradation is already at v_max_ with D-2-HG spike added on endogenous D-2-HG resulting in c(2-HG) > 200 µM. In contrast, formation of D-2-HG still increases upon increasing the concentration of α-ketoglutarate from 1 mM to 10 mM, proving a high D-2-HG production capacity. Again, endogenous D-2-HG raises the starting level in the 2-HG production assay. (**c**) Reaction velocity as a function of αKG-concentrations normalized to total protein for the HCT116 cell panel (wild type *IDH* and different *IDH1/2* mutations).

Pusch *et al*.^21^ had determined for IDH1-R132H (expressed and purified from *E. coli*) a K_m_ of 243.7 µM using αKG as a substrate. To compare *in vitro* D-2-HG production by mutated IDH1/2 and D-2-HG degradation by D2HDH, we performed both assays with pellets harvested from the same cell passage using an excess of the corresponding substrate and the endogenous redox equivalents. The D2HDH assay was performed in the presence 2 mM FAD. For the determination of IDH activity, we adapted the assay from Pusch *et al*.^21^ using a Tris-HCl buffer for cell lysis by sonication and quantifying 2-HG production over time after the addition of 1 mM to 10 mM α-ketoglutarate and 10 mM NADPH. Rate of D-2-HG production determined with this assay matched quite well intra- and extracellular 2-HG concentrations for the HCT116 panel (see Supplementary Table S3). 2-HG production in the parental cell line was found to be lowest, while HCT116 IDH1-R132H showed the highest 2-HG production rate as well as cellular levels (see Fig. 6c).

Compared to IDH, which is a low affinity/high capacity enzyme, D2HDH is a high affinity/low capacity enzyme. Therefore, D-2-HG degradation can already be measured at low D-2-HG concentrations, whereas for D-2-HG production high amounts of αKG have to be added to detect D-2-HG formation. On the other hand, maximum enzyme activity is reached at lower substrate concentrations of ~200 µM D-2-HG for D2HDH. However, when incubating cell homogenates without adding exogenous redox equivalents, D-2-HG concentration does not change significantly. Hence, we conclude that D-2-HG production and degradation do not impact each other when performing the respective assay. Running both assays at ~v_max_ clearly demonstrated that D-2-HG production exceeded D-2-HG degradation (see Fig. 6a,b, Table 1), resulting in an accumulation of D-2-HG as observed *in vivo*.

**Table 1:** Degradation rates calculated from data shown in Fig. 6a,b.

Cell line	Substrate spike level	2-HG in lysate [µM]	nmol 2-HG/(mg total protein × h)
**2-HG degradation**			**(n = 2)**
HCT116-IDH1R132H	100 µM D-2-HG	260.0	1.66
HCT116-IDH2R172K	100 µM D-2-HG	206.5	0.83
**2-HG production**			**(n = 2–4)**
HCT116-IDH1R132H	1 mM αKG	103.6	22.5
HCT116-IDH1R132H	10 mM αKG	113.6	28.5
HCT116-IDH2R172K	1 mM αKG	136.4	5.4
HCT116-IDH2R172K	10 mM αKG	151.6	15.3

Baseline 2-HG levels in samples used for determination of 2-HG formation is due to endogenous 2-HG in cells harbouring an *IDH* mutation.

## Discussion

Elevated D-2-HG levels impact prognosis and therapy of patients with mutated *IDH1/2* tumours like glioma or AML. They are also problematical for HG-aciduria patients. 2-HG is involved in the development and progression of these diseases and antibodies targeting mutated IDH1/2 in cancer can prolong survival in treated patients^29,30^. However, only for 2-HG-aciduria patients the mechanism of impaired 2-HG elimination is known. For *IDH1/2* mutant tumours, it has been only speculated that D-2-HG production is higher than D-2-HG degradation^19^. However, this assumption is based on k_cat_ of D2HDH in *Arabidopsis thaliana*, which was compared to human mutated IDH. In addition to K_m_ = 243.7 µM for IDH1-R132H from Pusch *et al*.^21^, Dang *et al*.^5^ determined the K_m_ of hIDH1-R132H expressed and purified from *E. coli* to be at 965 µM (for αKG as substrate). Conversely, both groups were not able to identify D-2-HG production by wild type IDH1. Though these two K_m_-values for IDH1-R132H do not completely agree, they show the same effect of an increased D-2-HG production by IDH1-R132H compared to wild type IDH1^5^. Nevertheless, it was not known how functional human D2HDH can deal with elevated D-2-HG levels. We could show that treatment of MCF7 cells with D-2-HG increased neither *D2HGDH* mRNA and D2HDH protein abundance nor D-2-HG degradation in comparison to untreated cells. Similarly, in HCT116 cells with endogenous D-2-HG, produced from mutated IDH1/2, degradation capacity was not drastically increased, though our data show high variation here. In addition, D2HDH protein was only significantly upregulated in HCT116 IDH2-R140Q in comparison to parental HCT116 with a 1.19-fold increase in relative abundance.

Here, we compared directly production and degradation of D-2-HG by mutated IDH1/2 and D2HDH, respectively. Regarding D-2-HG degradation, the K_m_ of 26.4 µM determined by our assay in the human breast adenocarcinoma cell MCF7 clearly exceeded the K_m_ values of 4.3 µM and 3.2 µM reported previously for human fibroblasts^25^ and rat liver^12^. Nevertheless, K_m_ values for D2HDH in mammalian cells are obviously much lower than the K_m_ value of ~580 µM reported for D2HDH from *Arabidopsis thaliana*^31^. Low K_m_ values indicate a high affinity of the enzyme to its substrate, which is typical for low concentrated substrates such as D-2-HG in healthy humans. This is also in line with the general suggestion of D2HDH being a metabolic repair enzyme^18,32^. Expression of D2HDH protein varied slightly between the cell lines tested here. Nevertheless, expression differences did not correlate with D-2-HG levels. For instance, Han *et al*. found that *D2HGDH* expression is upregulated upon HIF1α-stabilization^33^. However, it was also reported that HIF1α/-2α levels varied depending on the cell passage number^34^. In addition, it has been supposed that D-2-HG destabilizes HIF1^34,35^. Therefore, current knowledge is quite controversial with a lot of players impacting on each other. Perhaps this also contributed to the high variation in our D2HDH-assay data for the HCT116 panel. Further, from Table 1 it is obvious that the rates of production of D-2-HG by mutated IDH1/2 clearly exceeded those of its degradation by D2HDH when measured at maximum enzyme saturation (v_max_) and accounting for differences in protein expression of the enzymes.

For D-2-HG degradation, enzyme saturation might reflect physiological conditions with D2HDH being a high affinity/low capacity enzyme. However, for mutated IDH1/2 it is hard to evaluate how closely *in vitro* conditions mimic intracellular conditions. For mutated IDH1, it is known that D-2-HG production is limited by substrate availability^28^ and relies on intact wild type IDH1 activity, which can sufficiently refuel αKG. With regard to the close spatial proximity, one might speculate that local αKG concentrations are high enough for D-2-HG production at v_max_. For mutated IDH2, substrate availability is not an issue. It is speculated that there is effective regeneration of αKG by a network of enzymes, which tightly regulate mitochondrial αKG-levels via anaplerosis. For instance, there is conversion of glutamate to αKG in mitochondria (GLUD1/2, GOT2) and even in the cytosol (GOT1).

However, *in vivo* further events like substrate inhibition and export might affect net D-2-HG degradation and support negative D-2-HG mediated effects. Accordingly, Gelman *et al*. could not find many intracellular metabolic products originating from 2-HG degradation when attempting to track the fate of U-^13^C-2-HG administered to the HCT116 cell panel^36^. Moreover, with regard to the effect of D-2-HG on immune cells and, consequently, on immune response^35,37,38^, D2HDH activity of different types of immune cells might be worth studying.

In conclusion, our observations confirm that D-2-HG degradation is rather slow in comparison to its production in cells harbouring mutant *IDH*. This explains especially the high accumulation of D-2-HG in glioma tissue^5^, where there is no export into and distribution across other body fluids. Furthermore, our experiments show that D2HDH activity is different across the cell lines tested here. There are hints that expression of D2HDH is upregulated in the presence of high endogenous concentrations of D-2-HG.

## Supplementary information

Supplementary File

## Data Availability

The datasets generated during and/or analysed during the current study are available from the corresponding author on reasonable request.
