# Supplementary material for: Degradation of D-2-hydroxyglutarate in the presence of isocitrate dehydrogenase mutations
Source: Sci Rep. 2019 May 15;9:7436. doi: 10.1038/s41598-019-43891-3 (PMC6520482; doi:10.1038/s41598-019-43891-3)
Supplement: Supplementary file 1 — Supplementary File [file 41598_2019_43891_MOESM1_ESM.docx]

Degradation of D-2-hydroxyglutarate in the presence of isocitrate dehydrogenase mutations

**Raffaela S. Berger^1^, Lisa Ellmann^1^, Joerg Reinders^1,2^, Marina Kreutz^3^, Thomas Stempfl^4^, Peter J. Oefner^1^, Katja Dettmer^1*^**

# Supplementary Information

**Supplementary Table S1.** Figures of merit for the LC-MS/MS-based quantification of 2-hydroxyglutarate (2‑HG) were calculated from a calibration curve of 2-HG prepared in water.

|  | 2-HG |
| --- | --- |
| LOD (S/N>3) | < 0.05 µM |
| LLOQ | 0.2 µM |
| ULOQ | 900 µM |
| r (for calibration curve) | > 0.9988 |
| Interal standard | 2,3,3-d_3_-2-HG |

**Supplementary Table S2.** Mean accuracy, precision, and recovery. Mean accuracy is calculated from blank medium spiked with 2‑HG in known concentrations. Precision is calculated as standard deviation from repeated injection. Percent recovery of 2-HG from RPMI1640 cell culture medium with and without FCS was calculated from measurements of 2-HG in both un-spiked medium and medium spiked with different known amounts of 2‑HG standard.

| spike levels | accuracy/ precision (n=5) | Recovery for cell culture medium with FCS (n=6) | Recovery for cell culture medium w/o FCS (n=6) |
| --- | --- | --- | --- |
| 0.5 µM | 97.8 %± 5.7 | 101.8 % | 107.7 % |
| 25 µM | 100.8 %± 1.5 | 118.6 % | 125.5 % |
| 500 µM | 97.7 %± 2.0 | 111.9 % | 120.1 % |

Supplementary Table S3. Intracellular 2-HG levels in cell lines used for D2HDH assay.

| **cell line** | ***IDH* genotype** | **nmol 2-HG/**  **mg protein ± RSD** |
| --- | --- | --- |
| MCF7 | *wt* | 0.042 ± 23.7 % (n=6) |
| C7H2 | *wt* | 0.0001 ± 11.9 % (n=3) |
| HT1080 | *IDH1 R132C/+* | 78.0 ± 37.9 % (n=5) |
| HCT116 | *wt* | 0.17 ± 25.4 % (n=6) |
| HCT116 | *IDH1 R132H/+* | 46.60 ± 9.9 % (n=4) |
| HCT116 | *IDH2 R172K/+* | 28.36 ± 7.5 % (n=7) |
| HCT116 | *IDH2 R140Q/+* | 7.44 ± 12.0 % (n=6) |

Supplementary Table S4. Fold changes in D2HDH protein levels including significant p-values.

|  | MW relative protein abundance | fold change to HCT116 parental | p-value (TukeyHSD) < 0.05 | | | | | | |
| --- | --- | --- | --- | --- | --- | --- | --- | --- | --- |
|  |  |  | MCF7 | C7H2 | HCT116 parental | HCT116 IDH1-R132H | HCT116 IDH2-R172K | HCT116 IDH2-R140Q | HT1080 |
| MCF7 | 1.0 |  | - |  |  |  |  |  |  |
| C7H2 | 0.65 |  |  | - |  |  |  |  |  |
| HCT116 parental | 1.38 | 1.0 | 0.0104 | 1.20*10^-6^ | - |  |  |  |  |
| HCT116 IDH1-R132H | 1.61 | 1.17 | 5.92*10^-5^ | 1.87*10^-8^ |  | - |  |  |  |
| HCT116 IDH2-R172K | 1.58 | 1.15 | 7.44*10^-5^ | 1.78*10^-8^ |  |  | - |  |  |
| HCT116 IDH2-R140Q | 1.64 | 1.19 | 1.64*10^-5^ | 5.00*10^-9^ | 0.0350 |  |  | - |  |
| HT1080 | 1.45 |  | 0.0108 | 4.90*10^-6^ |  |  |  |  | - |

**Supplementary Table S5.** LC gradient for 2-HG quantification method

| **Time [min]** | **Flow rate [µL/min]** | **% Buffer A: water+0.1% FA** | **% Buffer B: ACN** |
| --- | --- | --- | --- |
| 0.0 | 200 | 100 | 0 |
| 6.5 | 200 | 100 | 0 |
| 8.0 | 350 | 0 | 100 |
| 10.0 | 350 | 0 | 100 |
| 10.1 | 350 | 100 | 0 |
| 17.0 | 350 | 100 | 0 |
| 17.1 | 200 | 100 | 0 |
| 18.0 | 200 | 100 | 0 |


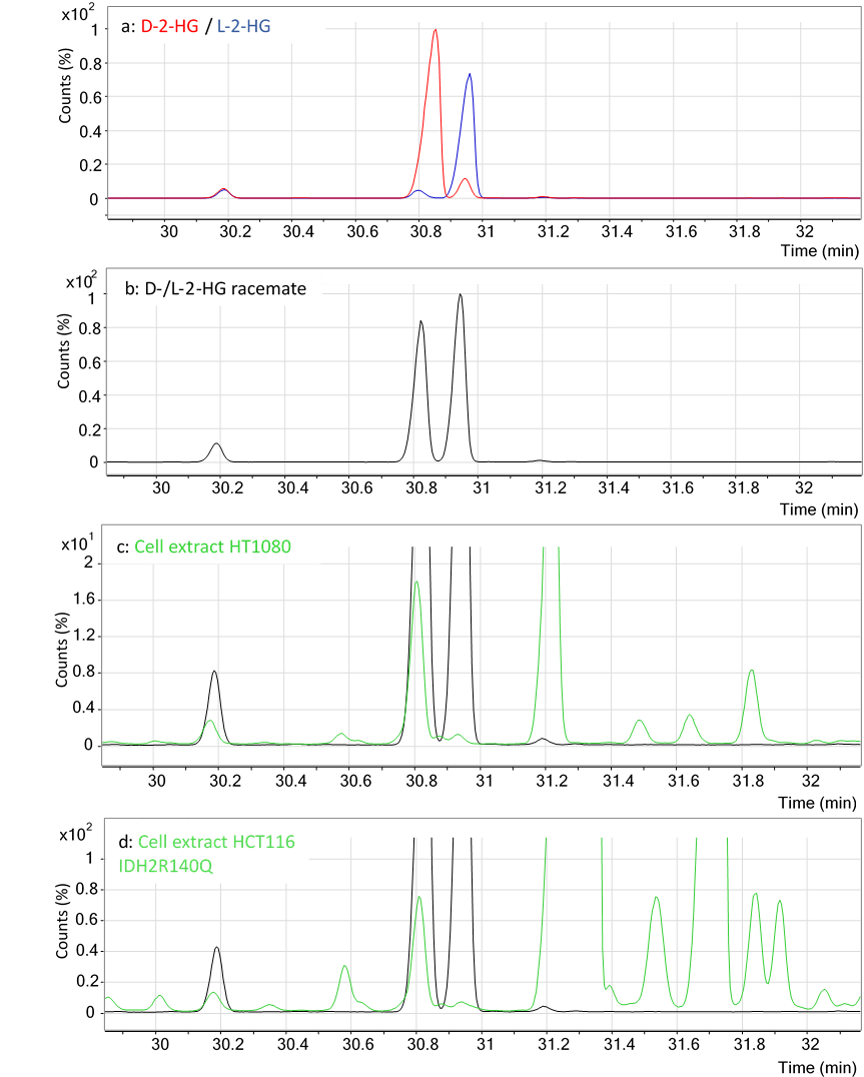


Supplementary Figure S1: Enantioselective analysis of 2-HG standards and cell extracts after chiral derivatization. (A) Chromatograms of separate D-2-HG (red) and L-2-HG (blue) standards. B) Chromatogram of a racemic standard of 2-HG. C) Chromatogram of an extract of HT1080 cells and D) HCT116 IDH2-R140Q cells (each overlaid with a chromatogram of the racemic standard in black). The enantioselective measurements clearly show, that in cell extracts mainly D-2-HG (left peak) is detected, but hardly any L-2-HG (right peak).

Enantioselective analysis was performed by GC-MS after chiral derivatization adapting a protocol by Gibson *et al*^1^. Briefly, standard solutions or biological samples were dried under a nitrogen stream. The residues were redissolved in 50 µL of (S)-(+)-2-butanol (Sigma Aldrich), acidified with 5 µL of 12 M HCl, and heated for 3 h at 90°C h. Samples were extracted with 500 µL of hexane and dried under nitrogen. For acetylation, 30 µL of pyridine and 30 µL of acetic anhydride were added to the residue and incubated for 1 h at 80°C. Again, samples were dried under nitrogen and redissolved in 50-100 µL of hexane. Analyses were performed on an Agilent 6890N GC equipped with a Mass Selective Detector Model 5975 Inert using a DB-Wax UI (Agilent; 30 m × 0.25 mm ID × 0.25 mm film thickness) column. The following parameters were used: injection volume 1 µL in splitless mode, helium flow 1.0 mL/min, full scan mode from 50–600 m/z. The initial oven temperature was set at 50°C, ramped at 5°C/min to 245°C, and held for 10 min.


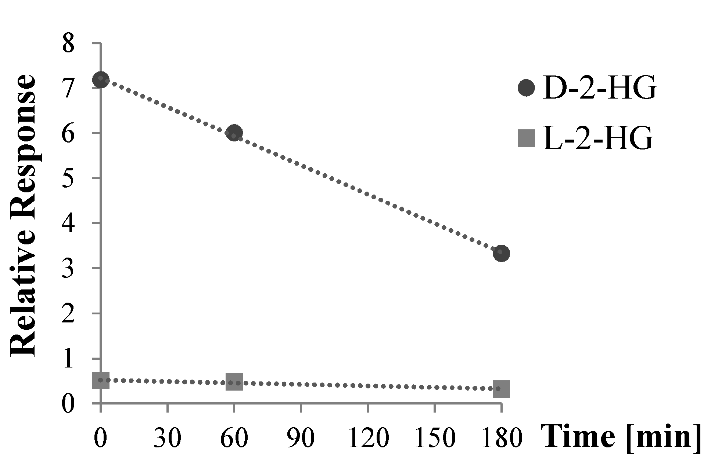


Supplementary Figure S2: Enantioselective measurement of enzyme assay aliquots for determination of D‑2‑HG degradation in an MCF7 cell homogenate. The assay was started by the addition of D‑2‑HG (>95% purity). Data points represent three replicate aliquots, which were pooled for the enantioselective analysis to receive a signal higher than the LOD. Degradation rate of D‑2‑HG agrees well with 2‑HG data from LC-MS/MS analysis.


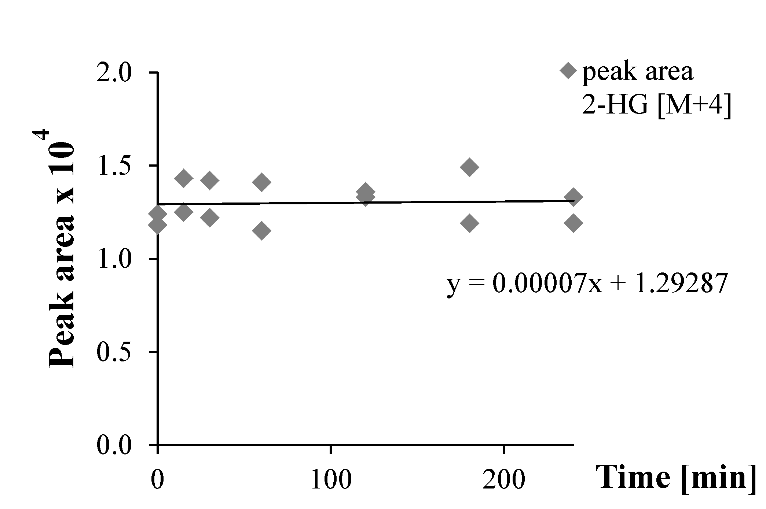


Supplementary Figure S3: Formation of 2-HG from α-ketoglutarate in the D2HGH-assay. To exclude formation of 2-HG from α-ketoglutarate by mutated IDH during assaying D-2-HG degradation by D2HGH, uniformly deuterated α-KG was incubated in an HT1080 cell homogenate. The plot shows peak areas corresponding to 2‑HG‑D4 over time. The values represent background levels, but do not increase significantly. Thus, 2-HG formation is ruled out.


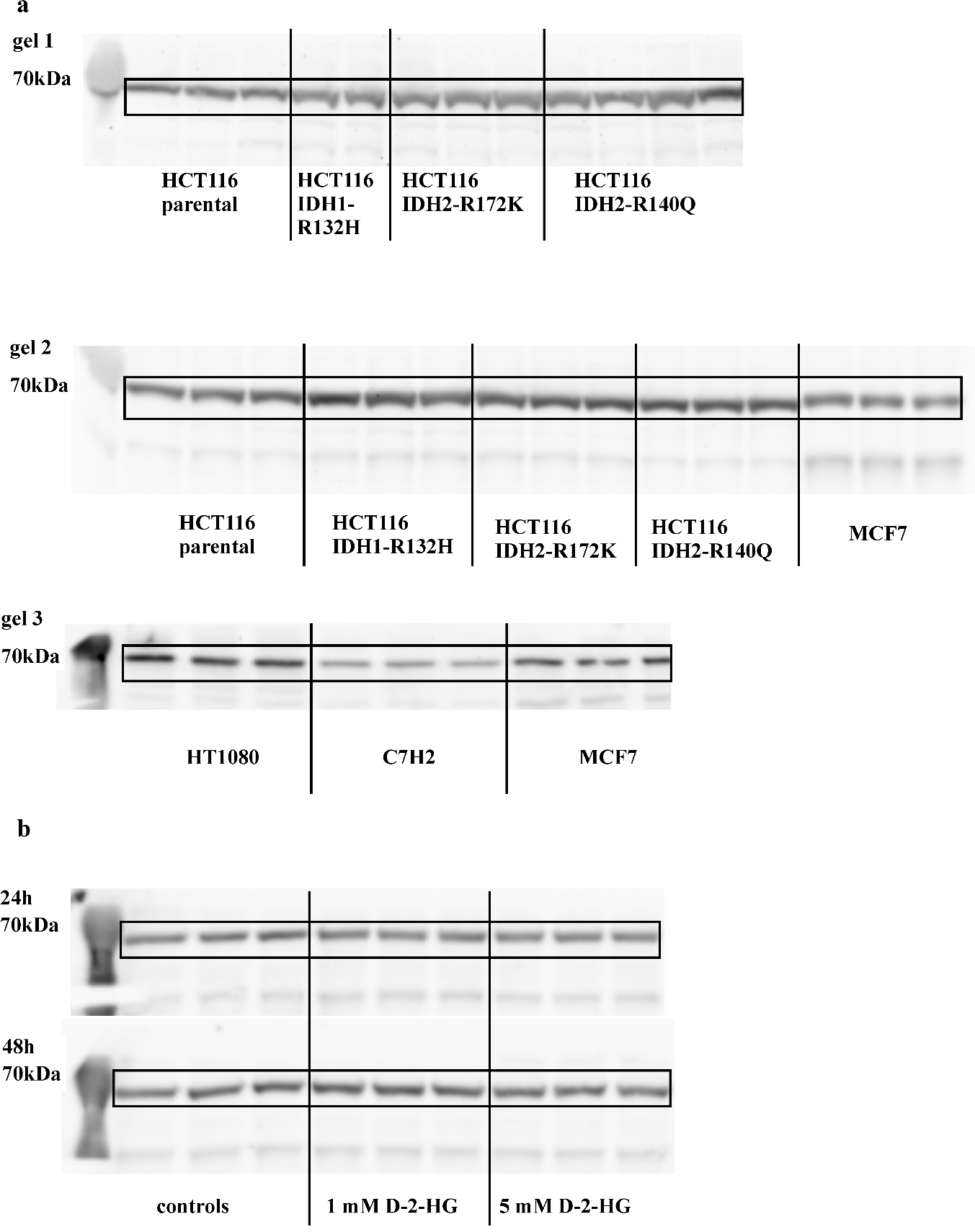


**Supplementary Figure S4**. Western blots for determination of D2HDH abundance in (**A**) the HCT116 panel, HT1080, C7H2, and MCF7, respectively. These blots are used to calculate relative abundance for Figure 3c (gel 3) and Figure 5c (gel 1+2). (**B**) D2HDH protein abundance in MCF7 after D-2-HG treatment. Gels were only blotted for the region of interest. Each lane represents an independent sample. Bands within the box were used for quantification using ImageLab-software.


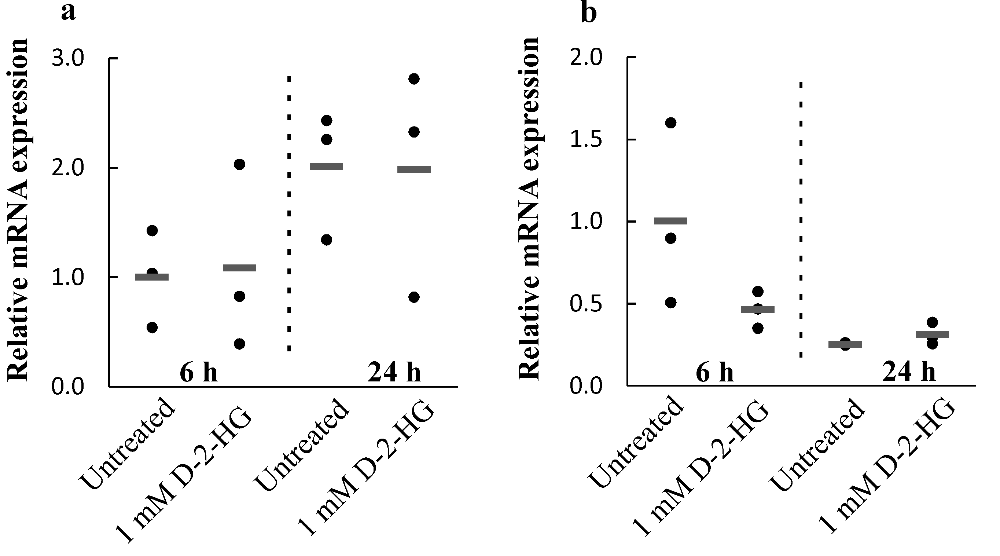


Supplementary Figure S5. Relative *D2HGDH* mRNA expression normalized to 18S rRNA (A) in MCF7 cells and (B) C7H2 cells untreated or treated with 1mM D-2-HG for 6 h and 24 h, respectively (n=3). Differences are not significant.





Supplementary Figure S6. Comparison of D2HDH enzyme activities between cells of the HCT116 panel and MCF7 cells. Variance in HCT116 is high, thus rendering the fitting of data obtained for HCT116 IDH2-R140Q impossible using the hill function.

**Reference**

# 1. Gibson, K. M. *et al*. Stable-isotope dilution analysis of D- and L-2-hydroxyglutaric acid: application to the detection and prenatal diagnosis of D- and L-2-hydroxyglutaric acidemias. *Pediatr. Res.* **34**, 277-280 (1993).
